# MASAN: a novel staging system for prognosis of patients with oesophageal squamous cell carcinoma

**DOI:** 10.1038/s41416-018-0094-x

**Published:** 2018-05-16

**Authors:** Wei Liu, Jian-zhong He, Shao-hong Wang, De-kai Liu, Xue-feng Bai, Xiu-e Xu, Jian-yi Wu, Yong Jiang, Chun-quan Li, Long-qi Chen, En-min Li, Li-yan Xu

**Affiliations:** 1 The Key Laboratory of Molecular Biology for High Cancer Incidence Coastal Chaoshan Area, Shantou University Medical College, Shantou, 515041 China; 2 Department of Mathematics, Heilongjiang Institute of Technology, Harbin, 150050 China; 3 Institute of Oncologic Pathology, Shantou University Medical College, Shantou, 515041 China; 4 Department of Pathology, Shantou Central Hospital, Affiliated Shantou Hospital of Sun Yat-Sen University, Shantou, 515041 China; 5 Department of Biochemistry and Molecular Biology, Shantou University Medical College, Shantou, 515041 China; 6 Department of Medical Informatics, Harbin Medical University-Daqing, Daqing, 163319 China; 7 Department of Thoracic Surgery, West China Hospital of Sichuan University, Chengdu, 610041 China

**Keywords:** Prognostic markers, Oesophageal cancer

## Abstract

**Background:**

Oesophageal squamous cell carcinoma (ESCC) is one of the most malignant cancers worldwide. Treatment of ESCC is in progress through accurate staging and risk assessment of patients. The emergence of potential molecular markers inspired us to construct novel staging systems with better accuracy by incorporating molecular markers.

**Methods:**

We measured H scores of 23 protein markers and analysed eight clinical factors of 77 ESCC patients in a training set, from which we identified an optimal MASAN (MYC, ANO1, SLC52A3, Age and N-stage) signature. We constructed MASAN models using Cox PH models, and created MASAN-staging systems based on k-means clustering and minimum-distance classifier. MASAN was validated in a test set (*n* = 77) and an independent validation set (*n* = 150).

**Results:**

MASAN possessed high predictive accuracies and stratified ESCC patients into three prognostic groups that were more accurate than the current pTNM-staging system for both overall survival and disease-free survival. To facilitate clinical utilisation, we also constructed MASAN-SI staging systems based on staining indices (SI) of protein markers, which possessed similar prognostic performance as MASAN.

**Conclusion:**

MASAN provides a good alternative staging system for ESCC prognosis with a high precision using a simple model.

## Introduction

Oesophageal squamous cell carcinoma (ESCC) is the fourth leading cause of cancer-related mortality, and approximately half of the world’s 500,000 new ESCC cases occur annually in China.^1, 2^ The survival for ESCC is poor, with a 5-year overall survival (OS) of 20.9%.^3^ Treatment of ESCC remains a challenging problem. However, treatment outcomes are being improved through accurate staging and risk assessment of patients.^4, 5^ Accurate staging techniques, including molecular staging, allow us to understand prognosis and to tailor therapy to individuals to achieve the best outcomes.

Currently, the most commonly used staging systems for ESCC is the pTNM (pathological tumour-node metastasis) staging system (the 7th edition) proposed by the American Joint Committee on Cancer (AJCC).^6^ The AJCC pTNM system has become a standardised staging system for evaluating cancer at a population level. However, the development of molecular biology and discovery of molecular factors that predict cancer outcome and response to treatment with better accuracy has led cancer experts to question the utility of the pTNM-staging system at the individual level.^7^ Molecular factors, such as protein markers, are attracting more and more attention and have been demonstrated to benefit the diagnosis and prognosis of ESCC. Incorporating molecular factors into predictive models may further improve the accuracy of the staging system.

Over the past few decades, hundreds of dysregulated proteins have been detected in ESCC patients.^8^ Many of them were identified to be independent prognostic factors, such as MYC,^9^ ANO1^10^ and ATF3.^11^ On the other hand, some clinical characteristics, such as N-stage, have always been predominant prognostic factors for ESCC.^12, 13^ Thus, Tan et al. proposed to combine protein markers and clinical characteristics, and built a FENSAM-staging system, which possessed high-classification precision similar to the pTNM-staging system, but was much simpler for clinical use.^14^ However, the protein markers used to build FENSAM were still limited. The predictive power of combinations of additional newly found protein markers needs further investigation. In addition, with more and more variables available for building predictive models, the anticipated predictive performance may not increase linearly with the number of variables due to complex interactions among variables.^15^ How to select an optimal feature combination and build robust predictive models remains a challenging problem.

To address this problem, we examine the expression of 23 potential protein markers and eight clinical characteristics of 304 ESCC patients, and propose a novel pipeline to identify optimal feature combination for model construction. We show that the resulting MASAN-staging system yields better prognostic capability than that of the pTNM-staging system, and provides a good alternative for clinical utilisation.

## Materials and methods

### Patients and specimens

Two independent data sets of formalin-fixed, paraffin-embedded tissue specimens were obtained from ESCC patients undergoing curative resection at the Shantou Central Hospital. The first data set included 154 patients treated during November 2007 to January 2010, and was randomly divided into a training set (*n* = 77) and a test set (*n* = 77). The clinicopathological characteristics were comparable in these two sets (Table 1). The training set was used to construct the predictive model and test set to evaluate the predictive performance. A second independent data set included 150 patients treated during 2000–2006 (validation set). All specimens were confirmed as ESCC by pathologists in the Clinical Pathology Department of the hospital, and the cases were classified according to the seventh edition of the AJCC pTNM system^6^ based on surgical T-stage, N-stage and M-stage. The surgical histologic grade of tumour differentiation was based on histological criteria of the guidelines of the WHO Classification of Tumours.^16^ Ethical approval was obtained from the ethical committee of the Central Hospital of Shantou City and the ethical committee of the Medical College of Shantou University. Only resected samples from surgical patients with written informed consent were included.

**Table 1:** Clinical characteristics of patients with ESCC in three data sets

Characteristics	Training set	Test set	*P* value^a^	Validation set	*P* value
No. of samples	77	77		150	
Age (median)	56	58	0.3721*	58	0.5866*
Gender			0.2035		0.8741
Male	60	67		114	
Female	17	10		36	
Smoking			0.1983		
Yes	53	61			
No	24	16			
Alcohol			0.3200		
Yes	26	33			
No	51	44			
Treatment			0.9612		4.07e-11
Surgery	37	39		131	
Surgery + chemotherapy	14	12		15	
Surgery + radiotherapy	18	17		4	
Surgery + chemoradiotherapy	8	9		0	
Tumour location			0.1496		0.1408
Upper	5	4		10	
Middle	44	33		104	
Lower	28	40		36	
Histologic grade			0.3035		0.0189
G1	12	9		43	
G2	61	59		91	
G3	4	9		16	
T-stage			0.1599		1.10e-05
T1	4	0		0	
T2	17	16		7	
T3	56	60		142	
T4	0	1		1	
N-stage			0.2119		0.0002
N0	33	40		76	
N1	21	25		61	
N2	16	8		12	
N3	7	4		1	
pTNM stage			0.6150		0.4284
I	3	5		2	
II	36	39		76	
III	38	33		72	
Death at follow-up			0.6124		0.0135
Yes	52	48		74	
No	25	29		76	
Overall survival, median (days)	1024	908	0.6751*	986	0.9167*
Disease-free status			1		0.0685
Yes	53	52		83	
No	24	25		67	
Disease-free survival, median (days)	648	683	0.9841*	974	0.1113*

^a^ *P* values were calculated by the *χ*^2^-test or Fisher’s exact test, unless otherwise stated. *Wilcoxon rank-sum test

### Tissue microarrays and immunohistochemistry

**Fig. 1:**
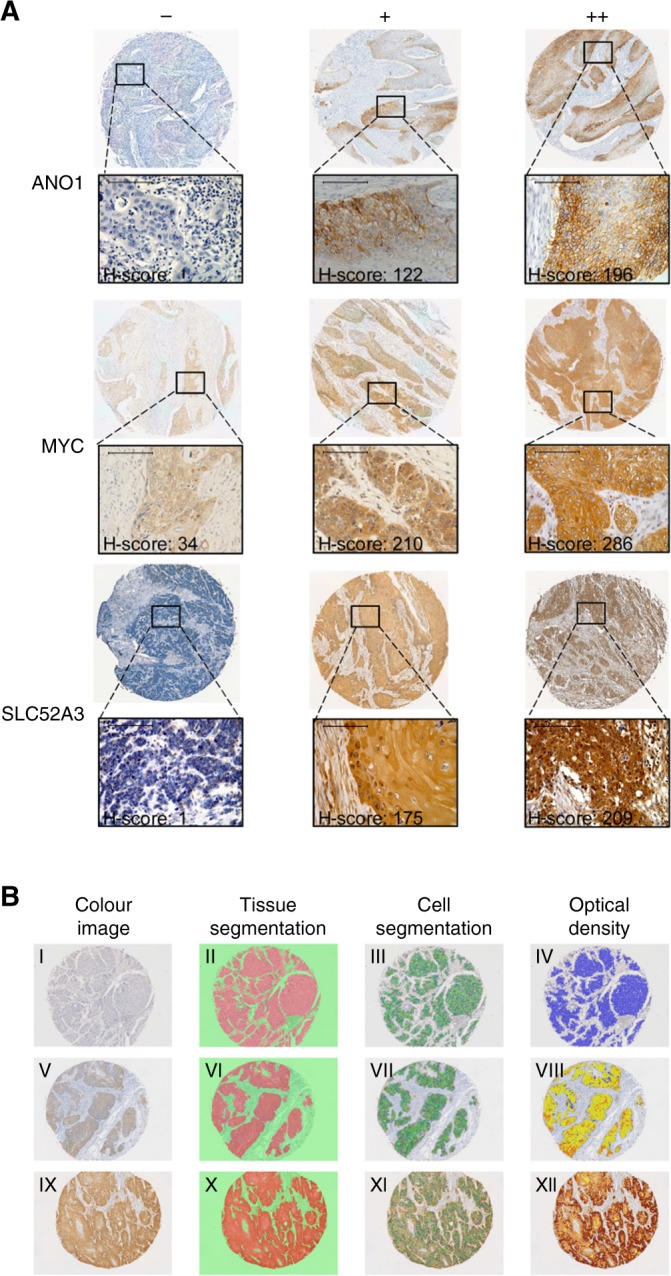
Representative images of IHC staining and scoring process. (**a**) Expression of ANO1, MYC, and SLC52A3 in TMAs. -: represents cases with negative or weak staining;+: represents cases with moderate staining;++: represents cases with intense staining base on manual assessment. H score represents the protein expression value of the corresponding case, evaluated by an automated quantitative pathology imaging system. (scale bars = 50 μm) (**b**) Scoring process: tissue, cell segmentation and spectral analysis by inform software. I, V, IX, Colour image of sample. II, VI X, Region training analysis of sample superimposed on the colour image. Red: tumour region; green: other. III, VII, XI, Composite image of cell segmentation of the tumour region, nucleus shown in green and the cytoplasm for each cell are outlined in colour around the nucleus. IV, VIII, XII: Spectral analysis based on the optical density grouping into 4 tiers: blue: 0, yellow:+, orange:++, and brown:+++. I-IV, V-VIII and IX-XII are from the same cores of the TMAs

Tissue microarray (TMA) construction and immunohistochemistry (IHC) staining were based on standard techniques as previously described^17^ (see Supplementary methods). Twenty-three markers were measured in this study (Fig. 1a and Figure S1). The detailed information on primary antibodies is listed in Table S1.

### Evaluation of IHC variables

We scored protein expression using two methods: a newly emerged technology for extracting the H score automatically^18^ and the traditional manual assessment-staining index (SI; see Supplementary methods).

### Statistical analysis

The univariate and multivariate Cox proportional hazards (Cox PH) models were built using the R package 'survival'. The predictive performance of Cox PH models was assessed using the concordance index (C-index)^19^ and area under the time-dependent ROC curve (AUC),^20^ which were calculated using the R package 'survcomp'. The k-means clustering algorithm was used to build the MASAN-staging system. The risk scores (RS) of patients in the training set were clustered into three clusters, which corresponded to the three MASAN stages. The thresholds of the MASAN stage were determined by a minimum-distance classifier. The genetic algorithm used to select optimal feature combination was performed using the R package 'mlr'.

## Results

### Identification of a MASAN signature

To construct a precise survival prediction model, we collected nine clinical characteristics (Table S2) and measured the expression of 23 proteins of 304 ESCC patients from two independent cohorts (see Materials and methods). IHC analysis showed that the immunostaining patterns of the 23 biomarkers were varied (Fig. 1a and Figure S1).

**Fig. 2:**
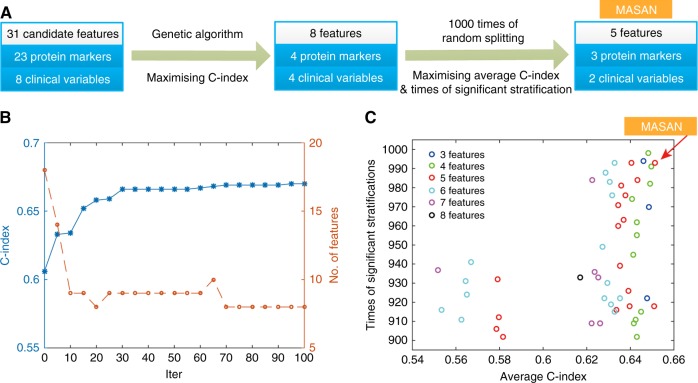
Construction of the MASAN model. **a** Pipeline of feature selection. **b** Procedure for optimisation of the genetic algorithm. Eight features (dotted yellow line) with a C-index (blue line) of 0.67 were identified at 100 iterations. **c** Comparison of predictive performance of all combinations of eight features. The combinations with times of significant stratification >900 were displayed. Five features (MASAN) with an average C-index of 0.6514 and 993 significant stratifications were identified

We designed a novel pipeline to identify optimal combinations of features (Fig. 2a). Initially, we used the genetic algorithm to select features from all 31 candidate features (23 proteins and 8 clinical variables) except pTNM stage. Eight features (fascin, MYC, ANO1, SLC52A3, age, smoking, G- and N-stage) with a C-index of 0.67 were identified after 100 iterations (Fig. 2b). Furthermore, an exhaustive search was performed to evaluate the predictive performance of all combinations of the eight features (Supplementary Methods). Feature combinations with both a high average C-index and a large number of times of significant stratification (located at the top right corner in Fig. 2c) were favourable signatures for survival prediction. Finally, five features (MYC, ANO1, SLC52A3, age and N-stage, MASAN) with an average C-index of 0.6514 and 993 significant stratifications were identified as the optimal feature combination (Fig. 2c).

### MASAN predicts the OS of ESCC patients

We constructed a Cox PH model using MASAN as independent variables and the OS information as dependent variables (referred to as MASAN model) from the training set (Table S3). The RS for OS (RS_os_) of a new patient *i* ($RS_{OS}^i$) can be calculated by formula (1):

1 $$\begin{array}{ccccc}\\ RS_{OS}^i{\mathrm{ = }} & 0.0027 \times \left( {E_{MYC}^i - 135.6169} \right) + 0.0094 \times \left( {E_{ANO1}^i - 13.2403} \right)\\ \\ & + 0.0032 \times \left( {E_{SLC52A3}^i - 59.5584} \right) + 0.0385 \times \left( {E_{Age}^i - 57.3117} \right)\\ \\ & + 0.6223 \times \left( {E_{N - stage}^i - 0.9610} \right)\\ \end{array}$$

where$E_{MYC}^i$,$E_{ANO1}^i$ and$E_{SLC52A3}^i$denote the H scores of MYC, ANO1 and SLC52A3, respectively. $E_{Age}^i$ and $E_{N - stage}^i$denote the age and N-stage of patient *i*, respectively.

To investigate the predictive ability of the MASAN model, we applied MASAN to predict RS_os_s of patients in the training set, test set and validation set, respectively. The RS_os_s yielded significant stratifications of patients, in all the three data sets, into low- and high-risk groups (*P* = 6.78 × 10^−4^, 1.07 × 10^−3^ and 7.57 × 10^−5^, respectively, Figure S2) using the median RS_os_ in the training set as the cutoff point, indicating that the predicted RS_os_s were quite consistent with the actual OS.

**Fig. 3:**
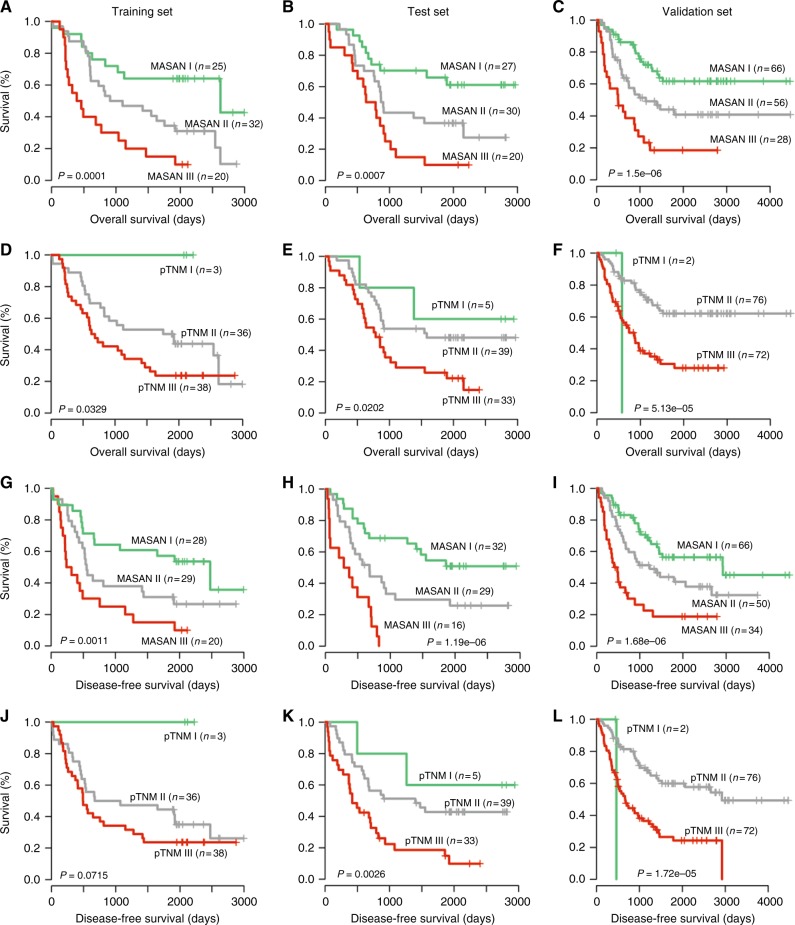
Comparison of the MASAN- and pTNM-staging systems on the OS and DFS of patients with ESCC by Kaplan–Meier analysis. **a**–**c** Kaplan–Meier curves using the MASAN system on OS for the training set (**a**), test set (**b**) and validation set (**c**). **d**–**f** Kaplan–Meier curves using the pTNM-staging system on OS for the training set (**d**), test set (**e**) and validation set (**f**). **g**–**i** Kaplan–Meier curves using the MASAN system on DFS for the training set (**g**), test set (**h**) and validation set (**i**). **j**–**l** Kaplan–Meier curves using the pTNM-staging system on DFS for the training set (**j**), test set (**k**) and validation set (**l**). *P* values were calculated by log-rank test

**Fig. 4:**
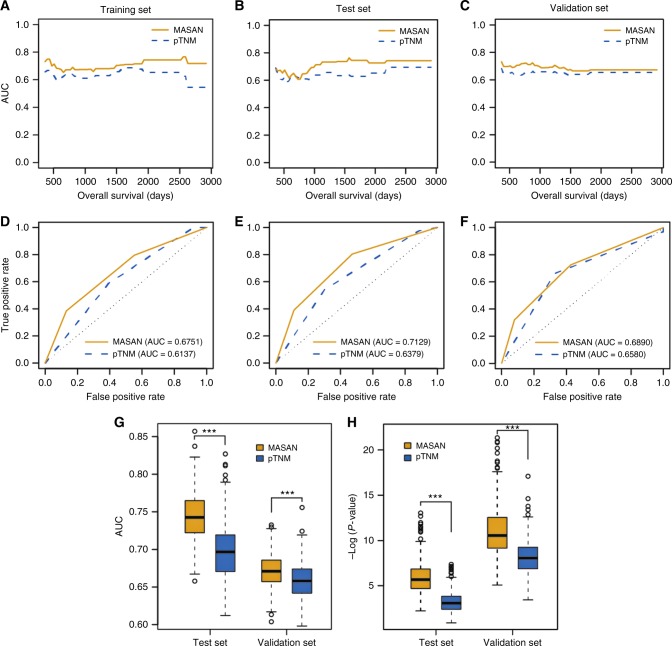
Predictive performance of the MASAN- and pTNM-staging systems on OS of patients with ESCC. **a**–**c** Time-dependent AUCs using the MASAN- and pTNM-staging systems on OS for the training set (**a**), test set (**b**) and validation set (**c**). **d**–**f** ROC curves of the MASAN- and pTNM-staging systems at the 3-year time point on OS for the training set (**d**), test set (**e**) and validation set (**f**). **g** Boxplots of AUCs using the MASAN- and pTNM-staging systems on the test set and validation set. ***P < 2.2 × 10^−16^. **h** Boxplots of –log (*P* values) using the MASAN and pTNM-staging systems on the test set and validation set. *P* values were calculated by the Wilcoxon-signed rank test

To compare the predictive ability of the MASAN model with the pTNM-staging system, we constructed a MASAN-staging system by clustering the patients in the training set into three groups using k-means clustering on the RS_os_s (Table S4). Kaplan–Meier analysis showed that the survival probabilities were significantly different among three stages (OS median = 1979, 1005.5 and 427 days for MASAN stages I–III, respectively, *P* = 0.0001, Fig. 4a). In contrast, the pTNM-staging system classified only three patients into stage I, and had a larger *P* value (*P* = 0.0329, Fig. 3d). The median AUC was larger for the MASAN than the pTNM system (0.7130 vs. 0.6432). In fact, the time-dependent AUCs for the MASAN-staging system were larger than those for the pTNM-staging system at each time point (Fig. 4a). Figure 4d shows the ROC curves for the two systems at the 3-year time point, where the superiority of the MASAN-staging system can be clearly observed.

Furthermore, the MASAN-staging system stratified the patients into three groups with significant OS differences for both the test set (*P* = 0.0007, Fig. 4b) and validation set (*P* = 1.5 × 10^−6^, Fig. 4c). In contrast, the stratifications of the pTNM-staging system had less significant OS differences (*P* = 0.0202 and 5.13 × 10^−5^, respectively, Fig. 3e,f). Specifically, the pTNM-staging system classified only a few patients into stage I for both the test set (*n* = 5) and the validation set (*n* = 2). The median AUC was larger for the MASAN than the pTNM-staging system (0.7332 vs. 0.6507 for the test set, and 0.6718 vs. 0.6555 for the validation set). Time-dependent AUC curves also showed that the MASAN-staging system yielded better predictive performance than that of the pTNM-staging system (Fig. 4b, c, e and f). Moreover, multivariable analysis showed that the MASAN signature was an independent prognostic factor for OS of ESCC patients in all three data sets (*P* = 0.0024, 0.0120 and 0.0022, respectively; Table S5).

In addition, to ensure that the predictive performance was not dependent on the particular patient set in the test set and validation set, we randomly chose 80% of patients from the two sets as the new test set (*n* = 61) and validation set (*n* = 120). Then we compared the predictive performance of the two systems on these two new sets by median AUC and *P* value of the log-rank test. We repeated the procedure 500 times. Boxplots showed that both the median AUCs and –log (*P* values) were significantly larger for the MASAN-staging system than the pTNM-staging system on the two new sets (Wilcoxon-signed rank test, *P* < 2.2 × 10^−16^ for all four comparisons, Fig. 4g, h). Besides, we also evaluated MASAN models on patients treated with surgery alone, and obtained similar prognostic performance (Figs. S3A and 3B). This further indicates that the MASAN-staging system is robust and produces consistently better ESCC prognosis.

### MASAN predicts DFS of ESCC patients

Next, we constructed a MASAN-staging system for DFS using the MASAN signature as independent variables, and the DFS information as dependent variables from the training set (Table S3). The RS for DFS (RS_DFS_) of a new patient *i* ($RS_{DFS}^i$) can be calculated by formula (2):

2 $$\begin{array}{ccccc}RS_{DFS}^i{\mathrm{ = }} & 0.0012 \times \left( {E_{MYC}^i - 135.6169} \right) + 0.0048 \times \left( {E_{ANO1}^i - 13.2403} \right)\\ & + 0.0057 \times \left( {E_{SLC52A3}^i - 59.5584} \right) + 0.0291 \times \left( {E_{Age}^i - 57.3117} \right)\\ & + 0.5856 \times \left( {E_{N - stage}^i - 0.9610} \right)\\ \end{array}$$

The predicted RS_DFS_s yielded significant stratifications of patients into low- and high-risk groups for the three data sets (*P* = 0.0011, 0.0037 and 6.18 × 10^−5^, respectively, Figure S4), indicating that the predicted RS_DFS_s were consistent with the actual DFS.

Next, we constructed the MASAN-staging system for DFS (Table S4). The MASAN-staging system again stratified the patients in three data sets into three stages with significant DFS differences (*P* = 1.1 × 10^−3^, 1.19 × 10^−6^ and 1.68 × 10^−6^, respectively, Fig. 3g-i). In contrast, the stratification with the pTNM-staging system was not significant for the training set (*P* = 0.0715, Fig. 3j) and less significant for the test set (*P* = 0.0026, Fig. 3k).

**Fig. 5:**
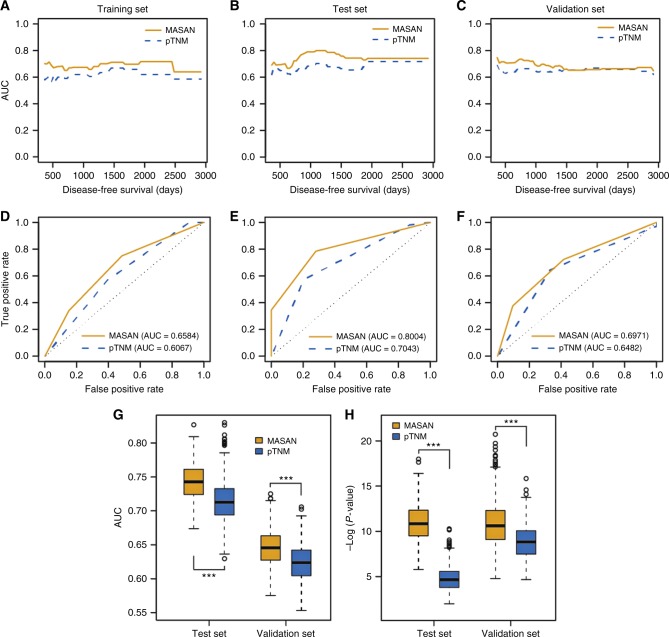
Predictive performance of the MASAN- and pTNM-staging systems on DFS of patients with ESCC. **a**–**c** The time-dependent AUCs of the MASAN- and pTNM-staging systems on DFS for the training set (**a**), test set (**b**) and validation set (**c**). **d**–**f** ROC curves of the MASAN- and pTNM-staging systems at the 3-year time point on DFS for the training set (**d**), test set (**e**) and validation set (**f**). **g** Boxplots of AUCs using the MASAN- and pTNM-staging systems on the test set and validation set. ***P < 2.2 × 10^−16^. **h** Boxplots of –log (*P* values) of the MASAN- and pTNM-staging systems on the test set and validation set. *P* values were calculated by the Wilcoxon-signed rank test

The median AUC was larger for the MASAN than the pTNM system for the three data sets (0.6972 vs. 0.6207, 0.7423 vs. 0.6827, and 0.6730 vs. 0.6542, respectively). Time-dependent AUC curves also showed that the MASAN system yielded better predictive performance than that of pTNM system (Figs. 5a-c and d–f). As OS, multivariable analysis of DFS showed that the MASAN signature was an in independent prognostic factor in all three data sets (*P* = 0.0093, 0.0002 and 0.0154, respectively; Table S5). And also, the MASAN-staging system had similar prognostic performance on patients treated with surgery alone (Figs. S3C and 3D). In addition, the permutation test also showed that the 500 AUCs and 500 –log (*P* values) were significantly larger for the MASAN-staging system than pTNM-staging system, respectively (Wilcoxon-signed rank test, *P* < 2.2 × 10^−16^ for all four comparisons, Fig. 5g, h).

### MASAN-SI predicts survival outcome of ESCC patients

For the convenience of clinical utilisation, we also constructed MASAN models using the SI of protein markers (MASAN-SI; Table S6). The RS for OS (RS-SI_OS_) and DFS (RS-SI_DFS_) of a new patient *i* can be calculated by formulae (3) and (4), respectively:

3 $$\begin{array}{ccccc}\\ RS{\mathrm{ - }}SI_{OS}^i{\mathrm{ = }} & 0.2662 \times \left( {SI_{MYC}^i - 1.0909} \right) + 0.6581 \times \left( {SI_{ANO1}^i - 0.0519} \right)\\ & + 0.2216 \times \left( {SI_{SLC52A3}^i - 0.3636} \right) + 0.0379 \times \left( {E_{Age}^i - 57.3117} \right)\\ & + 0.6063 \times \left( {E_{N - stage}^i - 0.9610} \right)\end{array}$$

4 $$\begin{array}{ccccc}RS{\mathrm{ - }}SI_{DFS}^i{\mathrm{ = }} & 0.1640 \times \left( {SI_{MYC}^i - 1.0909} \right) + 0.2697 \times \left( {SI_{ANO1}^i - 0.0519} \right)\\ & + 0.2483 \times \left( {SI_{SLC52A3}^i - 0.3636} \right) + 0.0293 \times \left( {E_{Age}^i - 57.3117} \right)\\ & + 0.5293 \times \left( {E_{N - stage}^i - 0.9610} \right)\end{array}$$

where $ST_{MYC}^i$,$ST_{ANO1}^i$ and$ST_{SLC52A3}^i$denote the SI of MYC, ANO1 and SLC52A3, respectively.

We constructed a MASAN-SI staging system using the thresholds listed in Table S7. Similar to the MASAN-staging system, MASAN-SI stratified ESCC patients into the three data sets into three stages with significant OS differences (*P* = 3.0 × 10^−4^, 6.0 × 10^−4^ and 2.0 × 10^−4^, respectively, Figure S5A-C) and DFS differences (*P* = 5.5 × 10^−3^, 2.05 × 10^−5^ and 9.55 × 10^−5^, respectively, Figure S5G-H). The time-dependent AUCs were larger for MASAN-SI- than the pTNM-staging system in the training set (OS: Figure S5D; DFS: Figure S5J) and test set (OS: Figure S5E; DFS: Figure S5K). In the validation set, the predictive performance of the two systems was comparable, with MASAN-SI slightly better on prognosis within 3 years (Figure S5F and S5L).

## Discussion

In this study, we examined the expressions of 23 potential protein markers and eight clinical characteristics of ESCC patients, from which we identified an optimal feature combination (MASAN) for precise prediction of ESCC survival outcome. We built MASAN models for both OS and DFS. The prognostic value of the MASAN models was verified in a test set and an independent validation set. Results showed that the MASAN-staging system yielded better prognostic performance than that of the pTNM-staging system.

The MASAN signature comprises both clinical factors and molecular factors. The clinical factors are essential as molecular factors alone could not accurately predict survival of ESCC patients (Figure S6A-C). In the MASAN model, coefficients are larger for N-stage than other features (formula (1)–(4)). Without N-stage, the prognostic performance was seriously deteriorated (Figure S6D-F). So N-stage is still a predominant prognostic factor, consistent with several previous studies.^12–14^ Positive expression of MYC and ANO1 has been found to be significantly correlated with poorer prognosis and suggested as potential biomarkers for ESCC patients.^9, 10^ In our three data sets, the expression values of ANO1 were high (>50) in only a small proportion of patients (6/77, 14/77 and 14/150, respectively). However, removing ANO1 from the MASAN model resulted in declined predictive performance, especially for DFS prediction in the validation set (Figure S6G), indicating that ANO1 plays a necessary role in the MASAN model. SLC52A3 has been suggested as a potential therapeutic target.^21^ Knockdown of SLC52A3 in ESCC cells results in inhibition of cell proliferation, whereas overexpression of SLC52A3 in ESCC cells promotes cell proliferation and tumourigenesis in nude mice.^21^ Age is also an essential factor in the MASAN model as removing age resulted in declined predictive performance (Figure S6H and 6I).

Beyond the superior predictive performance, the stratification of ESCC patients is more reasonable for MASAN-staging system than the pTNM-staging system. The MASAN-staging system stratifies more patients into the low-risk group compared to pTNM-staging system (Fig. 3). Furthermore, stratification by the MASAN-staging system possesses more consistent and higher OS for low-risk patients, and lower OS for high-risk patients, while pTNM fluctuated more widely (Table S8). DFS also had the same tendency (Table S9). Thus, the MASAN-staging system provides better guidance for making clinical decisions. More low-risk patients may avoid unnecessary treatments. Moreover, the MASAN model is based on protein markers and clinical characteristics, and is easy to use. On the basis of a simple model, MASAN provides a good alternative staging system for ESCC patients with a high precision.

Note that, although MASAN is reliable for Chinese patients, it must be careful to use it for prognosis of Caucasian patients as there exists differences between Asian and Caucasian patient populations in both clinicopathologic and molecular features.^22, 23^ The feasibility of MASAN or new staging models on Caucasian patients will be investigated when we have enough samples in future. Another limitation is that, as a retrospective study, the patients used in this study were mostly collected between 2000 and 2010, which lacked necessary pre-operative information for accurate clinical staging system. Thus, MASAN cannot be used as a clinical staging system. As clinical staging system is of great value for patient care, pre-operative information of ESCC patients should be included to construct novel clinical staging system with better accuracy in future.

To facilitate clinical utilisation, we constructed prognostic models using both H score (MASAN) and SI (MASAN-SI). Results show that MASAN-SI obtains similar prognostic performance as MASAN. Both models are available at http://www.licpathway.net/MASAN/index.php.

## Electronic supplementary material

Supplementary methods

Supplementary tables

Figure S1

Figure S2

Figure S3

Figure S4

Figure S5

Figure S6

Supplementary Figures
