# Supplementary material for: MASAN: a novel staging system for prognosis of patients with oesophageal squamous cell carcinoma
Source: Br J Cancer. 2018 May 16;118(11):1476–84. doi: 10.1038/s41416-018-0094-x (PMC5988697; doi:10.1038/s41416-018-0094-x)
Supplement: Supplementary file 1 — Supplementary methods [file 41416_2018_94_MOESM1_ESM.docx]

## Supplementary methods

#### The Cox proportional hazards (Cox PH) models

The Cox PH model estimates the hazard of patient *i* at time *t* by the formula (1):

$h\left(t,\mathbf{X}\right)=h_{0}\left(t\right)\exp\left(\beta_{1}X_{1}+\beta_{2}X_{2}+\text{…}+\beta_{p}X_{p}\right)=h_{0}\left(t\right)\exp\left(\mathbf{\beta}^{T}\mathbf{X}\right)$ (1)

where $\mathbf{X}=\left(X_{1},X_{2},\text{…,}X_{p}\right)^{T}$ is the vector of the features, $\mathbf{\beta}=\left(\beta_{1},\beta_{2},\text{…},\beta_{p}\right)^{T}$ is the vector of regression coefficients, $h_{0}\left(t\right)$ is an unspecified baseline hazard function, and the cumulative hazard function $h\left(t,\mathbf{X}\right)$ is a relative risk. So we used $\mathbf{\beta}^{T}\mathbf{X}$as the risk score (RS) to predict the survival outcome of patients.

#### TMAs construction and IHC staining

TMAs construction were based on standard techniques as previously described [^1^](#_ENREF_1), and IHC staining was carried out using the PV-9000 2-step Polymer Detection System (ZSGB-BIO, Beijing, China) and Liquid DAB Substrate Kit (Invitrogen, San Francisco, CA) according to the manufacturer’s instructions. In brief, the sections were deparaffinised, rehydrated, antigen retrieval. And then, 3% hydrogen peroxide was used to remove endogenous peroxidase activity at room temperature for 10 min. After washing in phosphate-buffered saline (PBS), 10% normal goat serum was used to block non-specific antibody; whereafter, sections were incubated overnight at 4^◦^C with primary antibodies. After rinsing in PBS, each section was incubated with PV-9000 2-step Polymer Detection System, and the primary antibodies were detected with Liquid DAB Substrate Kit. Sections were counterstained with hematoxylin after washing in PBS. Lastly, slides were dehydrated, cleared and mounted.

#### Evaluation of IHC variables

We scored protein expression using two methods: a newly emerged technology for extracting the H score automatically, and the traditional manual assessment-staining index (SI). H score was evaluated by an automated quantitative pathology imaging system (Perkin Elmer, Waltham, MA, USA), as described previously [^2^](#_ENREF_2). Briefly, we used Vectra 2.0.8 software for automated image acquisition, and obtained the colour images (Figure 1B, I, V, and IX). Subsequently, the spectral libraries were constructed using Nuance 3.0 software. The colour images were then evaluated by Inform 1.2 software following three steps: i) segmentation of the tumour region from the tissue compartments (Figure 1B, II, VI, and X); ii) segmentation of the cells from the tumour region (Figure 1B, III, VII, and XI); and iii) calculation of the H score based on the optical density. The H score (= (% at 0)*0+(% at 1+)*1+(% at 2+)*2+(% at 3+)*3) produces a continuous protein expression value in the range of 0 to 300 [^2^](#_ENREF_2).

To facilitate clinical application, we also evaluated protein expression using the SI measure. SI is a 3-tiered protein expression value with 0 representing “-” (negative/weak staining), 1 “+” (moderate staining), and 2 “++” (intense staining). It was determined based on both the intensity of staining and the proportion of tumour cells showing unequivocal positive staining according to the literature [^1^](#_ENREF_1). Each separate tissue core was scored by two independent histopathologists who were blinded to the clinical outcome. Discrepancies were resolved by consensus.

#### Genetic algorithm for feature selection

A genetic algorithm was used to select optimal feature combination with best predictive performance. We used “bit string” to encode each feature combination and “C-index” as the fitness function. We randomly generated an initial population (*size* = 50) of feature combinations. For each feature combination, we constructed a Cox PH model based on 80% of the patients in the training set, and calculated its C-index on the remaining 20% patients. To maximize the C-index, the genetic algorithm calculated the C-index for each feature combination in the current population, and generated a new generation population by selection, crossover, and mutation operators. This process was repeated until the maximal number of iterations was reached. The genetic algorithm was performed using the R package “mlr.” We set the maximal number of features as 15 and maximal number of iterations as 100, respectively. Other parameters were set following the recommended default values.

#### The exhaustive search to identify optimal feature combination

To identify a robust optimal feature combination, we randomly chose a subset of samples (80%) in the training set, and built Cox PH models on the subset, using all combinations of the eight features, respectively. The trained Cox PH models were used to predict risk scores of patients in the subset and stratified the patients into low- and high-risk groups by using the median risk score as the cutoff point. Then the predictive performance of feature combinations was evaluated by C-index on the subset and whether the stratification was significant (log-rank test, *P* < 0.05). This process was repeated 1000 times.

#### References

1. Xie JJ, Xu LY, Wu ZY, Zhao Q, Xu XE, Wu JY, et al. Prognostic implication of ezrin expression in esophageal squamous cell carcinoma. *J Surg Oncol* **104**, 538-543, (2011).

2. Huang W, Hennrick K, Drew S. A colorful future of quantitative pathology: validation of Vectra technology using chromogenic multiplexed immunohistochemistry and prostate tissue microarrays. *Hum Pathol* **44**, 29-38, (2013).
