# Supplementary material for: MASAN: a novel staging system for prognosis of patients with oesophageal squamous cell carcinoma
Source: Br J Cancer. 2018 May 16;118(11):1476–84. doi: 10.1038/s41416-018-0094-x (PMC5988697; doi:10.1038/s41416-018-0094-x)
Supplement: Supplementary file 2 — Supplementary tables [file 41416_2018_94_MOESM2_ESM.doc]

**Table S1. Primary antibodies used in this study.**

| Antibody | Species | Company (catalogue number) | Working concentration | Polymer Detection System |
| --- | --- | --- | --- | --- |
| ANO1 | Rabbit Monoclonal | LSBio (LS-C88846) | 1:50 | PV-9000 |
| Myc | Mouse Monoclonal | Santa Cruz Biotechnology | 1:100 | PV-9000 |
| Myc | Mouse Monoclonal | Santa Cruz Biotechnology | 1:100 | DAKO |
| SLC52A3 | Rabbit Polyclonal | Abgent | 1:50 | PV-9000 |
| SLC52A3a | Rabbit Polyclonal | ZhouShan biological technology, China | 1:10 | PV-9000 |
| SLC52A3b | Rabbit Polyclonal | ZhouShan biological technology, China | 1:50 | PV-9000 |
| CREPT | Mouse Monoclonal | Gift from Zhijie Chang | 1:50 | PV-9000 |
| Fascin | Mouse Monoclonal | Dako (M3567) | 1:100 | PV-9000 |
| P-Fascin | Rabbit Polyclonal | Beijing Biosynthesis Biotechnology, Beijing, China | 1:100 | PV-9000 |
| STMN1 | Mouse Monoclonal | Santa Cruz Biotechnology (sc-48362) | 1:50 | PV-9000 |
| HSPB1 | Mouse Monoclonal | Santa Cruz Biotechnology (sc-13132) | 1:2000 | PV-9000 |
| CNPY2 | Rabbit Polyclonal | Sigma (HPA038465) | 1:700 | PV-9000 |
| PDIA3 | Rabbit Polyclonal | Sigma (HPA002645) | 1:700 | PV-9000 |
| ATF3 | Mouse Polyclonal | Abgent (AT1223a) | 1:50 | PV-9000 |
| esVEGFR2 | Rabbit Polyclonal | Capital Bio-sciences (Beijing, China) | 1:50 | PV-9000 |
| Trkb | Rabbit Monoclonal | Sino biological (10047-R107) | 1:50 | PV-9000 |
| L2△e13 | Rabbit Polyclonal | Bioss Beijing, China | 1:500 | PV-9000 |
| ASNS |  | Gift from Dong Xie | 1:1000 | PV-9000 |
| BLNK | Rabbit Polyclonal | Sino biological (12706-RP02) | 1:50 | PV-9000 |
| DSC2 | Mouse Monoclonal | Santa Cruz Biotechnology (sc-53485) | 1:50 | PV-9000 |
| ITGB4 | Rabbit Polyclonal | Santa Cruz Biotechnology (sc-9090) | 1:200 | PV-9000 |
| ITGA6 | Rabbit Polyclonal | Santa Cruz Biotechnology (sc-10730) | 1:50 | PV-9000 |
| ITGA5B1 | Mouse Monoclonal | Millipore (MAB1969) | 1:50 | PV-9000 |
| Ezrin | Mouse Monoclonal | Neomarkers (MS-661) | 1:100 | PV-9000 |

**Table S2. Univariate analysis of protein markers and clinical characteristics in the training set.**

| **Variables** | **OS HR (95% CI)** | **OS *P*-value*** | **DFS HR (95% CI)** | **DFS *P*-value** | |
| --- | --- | --- | --- | --- | --- |
| ANO1 | 1.0013(0.9898-1.0129) | 0.8304 | 0.9988(0.9872-1.0106) | | 0.8443 |
| MYC | 1.0030(0.9994-1.0065) | 0.1027 | 1.0019(0.9984-1.0055) | | 0.2813 |
| SLC52A3 | 0.9977(0.9920-1.0035) | 0.4375 | 1.0003(0.9946-1.0059) | | 0.9258 |
| ASNS | 0.9992(0.9917-1.0069) | 0.8421 | 1.0013(0.9938-1.0089) | | 0.7264 |
| ATF3 | 0.9982(0.9942-1.0023) | 0.3916 | 0.9992(0.9952-1.0031) | | 0.6716 |
| Blnk | 1.0003(0.9945-1.0061) | 0.9295 | 0.9991(0.9934-1.0049) | | 0.7680 |
| CNPY2 | 0.9966(0.9915-1.0017) | 0.1885 | 0.9986(0.9938-1.0034) | | 0.5600 |
| CREPT | 0.9983(0.9924-1.0043) | 0.5839 | 0.9984(0.9926-1.0042) | | 0.5877 |
| DSC2 | 1.0013(0.9968-1.0057) | 0.5812 | 1.0011(0.9968-1.0055) | | 0.6163 |
| Ezrin | 0.9974(0.9915-1.0033) | 0.3843 | 0.9965(0.9911-1.0020) | | 0.2096 |
| Fascin | 1.0013(0.9961-1.0065) | 0.6349 | 1.0004(0.9954-1.0055) | | 0.8637 |
| P-Fascin | 0.9946(0.9891-1.0002) | 0.0609 | 0.9957(0.9905-1.0010) | | 0.1131 |
| HSPB1 | 0.9987(0.9951-1.0024) | 0.5042 | 0.9987(0.9952-1.0022) | | 0.4744 |
| ITGA6 | 0.9975(0.9916-1.0034) | 0.4036 | 0.9984(0.9926-1.0042) | | 0.5818 |
| ITGA4 | 0.9998(0.9939-1.0059) | 0.9590 | 1.0005(0.9947-1.0063) | | 0.8643 |
| ITGA5B1 | 1.0027(0.9947-1.0107) | 0.5102 | 1.0017(0.9939-1.0095) | | 0.6773 |
| L2△e13 | 0.9996(0.9945-1.0047) | 0.8797 | 0.9991(0.9942-1.0041) | | 0.7341 |
| PDIA3 | 0.9948(0.9892-1.0004) | 0.0693 | 0.9959(0.9904-1.0013) | | 0.1393 |
| SLC52A3a | 1.0016(0.9978-1.0053) | 0.4159 | 1.0016(0.9979-1.0052) | | 0.4015 |
| SLC52A3b | 0.9990(0.9941-1.0039) | 0.6802 | 0.9998(0.9949-1.0047) | | 0.9300 |
| STMN1 | 1.0002(0.9948-1.0057) | 0.9328 | 0.9992(0.9937-1.0048) | | 0.7872 |
| esVEGFR2 | 0.9997(0.9953-1.0042) | 0.9048 | 1.0003(0.9959-1.0046) | | 0.9079 |
| Trkb | 0.9999(0.9876-1.0123) | 0.9877 | 0.9979(0.9859-1.0101) | | 0.7363 |
| Gender | 0.9303(0.4877-1.7745) | 0.8263 | 0.8719(0.4581-1.6595) | | 0.6764 |
| Age | 1.0216(0.9936-1.0504) | 0.1323 | 1.0170(0.9898-1.0449) | | 0.2223 |
| Tumour location | 0.9957(0.6355-1.5601) | 0.9850 | 1.1497(0.7309-1.8086) | | 0.5460 |
| Smoke | 1.5207(0.8215-2.8149) | 0.1821 | 1.4001(0.7677-2.5534) | | 0.2723 |
| Alcohol | 2.3771(1.3405-4.2153) | **0.0030** | 1.8986(1.0868-3.3168) | | **0.0243** |
| Tumour grade | 1.1537(0.6140-2.1677) | 0.6568 | 1.1830(0.6540-2.1401) | | 0.5784 |
| N-stage | 1.5524(1.1777-2.0463) | **0.0018** | 1.4821(1.1204-1.9605) | | **0.0058** |
| T-stage | 2.0901(1.1346-3.8502) | **0.0180** | 2.1387(1.1685-3.9145) | | **0.0137** |
| pTNM stage | 1.9431(1.1670-3.2352) | **0.0107** | 1.7097(1.0474-2.7908) | | **0.0319** |

**P*-values are estimated by univariate Cox regression analysis.

**Table S3.** **Estimated coefficients for variables used in the MASAN model on OS and DFS.**

| **Variables** | **Mean^a^** | **Coefficients** | |
| --- | --- | --- | --- |
|  |  | OS | DFS |
| **MYC** | 135.6169 | 0.0027 | 0.0012 |
| **ANO1** | 13.2403 | 0.0094 | 0.0048 |
| **SLC52A3** | 59.5584 | 0.0032 | 0.0057 |
| **Age** | 57.3117 | 0.0385 | 0.0291 |
| **N-stage** | 0.9610 | 0.6233 | 0.5856 |

^a^Mean values of variables in the training set, which were used to center the variables when predicting the risk of new patients. OS, Overall survival; DFS, Disease-free survival; MASAN, MYC-ANO1-SLC52A3-Age-N stage

**Table S4. Thresholds of the MASAN grade for OS and DFS**.

| **MASAN grade** | **Risk score range** | |
| --- | --- | --- |
|  | OS | DFS |
| **I** | < -0.3461 | < -0.2578 |
| **II** | - 0.3461 ~ 0.4514 | -0.2578 ~ 0.3845 |
| **III** | > 0.4514 | > 0.3845 |

Abbreviation: OS, Overall survival; DFS, Disease-free survival; MASAN, MYC-ANO1-SLC52A3-Age-N stage

| | **Table S5. Univariate and multivariate analysis of factors associated with overall survival (OS) and disease-free survival (DFS) in three datasets.** | | | | | | | | | | | |  | | --- | --- | --- | --- | --- | --- | --- | --- | --- | --- | --- | --- | --- | | Variables | Univariate analysis | | | | |  | Multivariate analysis | | | | |  | | OS | |  | DFS | |  | OS |  |  | DFS |  |  | | HR(95%CI) | *P* |  | HR(95%CI) | *P* |  | HR(95%CI) | *P* |  | HR(95%CI) | *P* |  | | **Training set** |  |  |  |  |  |  |  |  |  |  |  |  | | Age | 1.0216(0.9936-1.0504) | 0.1323 |  | 1.0170(0.9898-1.0449) | 0.2223 |  |  |  |  |  |  |  | | Gender (Female/Male) | 0.9303(0.4877-1.7745) | 0.8263 |  | 0.8719(0.4581-1.6595) | 0.6764 |  |  |  |  |  |  |  | | Smoking (Y/N) | 1.5207(0.8215-2.8149) | 0.1821 |  | 1.4001(0.7677-2.5534) | 0.2723 |  |  |  |  |  |  |  | | Alcohol (Y/N) | 2.3771(1.3405-4.2153) | **0.0030** |  | 1.8986(1.0868-3.3168) | **0.0243** |  |  |  |  |  |  |  | | Treatment (surgery + Adjuvant therapy /surgery) | 0.9336(0.7224-1.207) | 0.5998 |  | 0.9390(0.7225-1.2200) | 0.6380 |  |  |  |  |  |  |  | | Tumour location (upper, middle, lower) | 0.9957(0.6355-1.5601) | 0.9850 |  | 1.1497(0.7309-1.8086) | 0.5460 |  |  |  |  |  |  |  | | Histologic grade(G1, G2, G3) | 1.1537(0.6140-2.1677) | 0.6568 |  | 1.1830(0.6540-2.1401) | 0.5784 |  |  |  |  |  |  |  | | T stage (T1, T2, T3, T4) | 2.0901(1.1346-3.8502) | **0.0180** |  | 2.1387(1.1685-3.9145) | **0.0137** |  |  |  |  |  |  |  | | N stage (N0, N1, N2, N3) | 1.5524(1.1777-2.0463) | **0.0018** |  | 1.4821(1.1204-1.9605) | **0.0058** |  |  |  |  |  |  |  | | pTNM stage (I, II, III) | 1.9431(1.1670-3.2352) | **0.0107** |  | 1.7097(1.0474-2.7908) | **0.0319** |  |  |  |  |  |  |  | | MASAN signature (I, II, III) | 2.2330(1.5160-3.2870) | **4.718×10^-05^** |  | 1.8938(1.3280-2.7000) | **0.0004** |  | 2.0456(1.2892-3.2460) | **0.0024** |  | 1.7419(1.1468-2.6460) | **0.0093** |  | | **Test set** |  |  |  |  |  |  |  |  |  |  |  |  | | Age | 1.0262(0.9969-1.0560) | 0.0806 |  | 1.0155(0.9877-1.0440) | 0.2780 |  |  |  |  |  |  |  | | Gender (Female/Male) | 0.8917(0.3786-2.1000) | 0.7932 |  | 1.1099(0.4736-2.6010) | 0.8103 |  |  |  |  |  |  |  | | Smoking (Y/N) | 0.9771(0.4864-1.9630) | 0.948 |  | 1.0329(0.5180-2.0600) | 0.9268 |  |  |  |  |  |  |  | | Alcohol (Y/N) | 1.0481(0.5897-1.8630) | 0.8729 |  | 0.8672(0.4957-1.5170) | 0.6176 |  |  |  |  |  |  |  | | Treatment (surgery + Adjuvant therapy /surgery) | 1.3170(1.0090-1.7190) | **0.0427** |  | 1.5330(1.1950-1.9670) | **0.0008** |  |  |  |  | 1.8830(1.0241-3.4640) | **0.0417** |  | | Tumour location (upper, middle, lower) | 0.7947(0.5028-1.2560) | 0.3251 |  | 0.9000(0.5678-1.4270) | 0.6540 |  |  |  |  |  |  |  | | Histologic grade(G1, G2, G3) | 2.7782(1.3340-5.7870) | **0.0063** |  | 3.4677(1.5630- 7.6950) | **0.0022** |  |  |  |  |  |  |  | | T stage (T1, T2, T3, T4) | 0.9966(0.5093-1.9500) | 0.9921 |  | 0.7906(0.4259-1.4670) | 0.4565 |  |  |  |  |  |  |  | | N stage (N0, N1, N2, N3) | 1.7183(1.2550-2.3530) | **0.0007** |  | 1.8241(1.3550-2.4560) | **7.523×10^-05^** |  |  |  |  |  |  |  | | pTNM stage (I, II, III) | 1.9788(1.1960-3.2740) | **0.0079** |  | 2.2624(1.3830-3.7010) | **0.0012** |  |  |  |  |  |  |  | | MASAN signature (I, II, III) | 2.0270(1.3880-2.9590) | **0.0002** |  | 2.4903(1.6950-3.6590) | **3.396×10^-05^** |  | 1.8590(1.1457-3.0160) | **0.012** |  | 2.4780(1.5446- 3.9750) | **0.0002** |  | | **Validation set** |  |  |  |  |  |  |  |  |  |  |  |  | | Age | 1.0152(0.9879-1.0430) | 0.2783 |  | 1.0163(0.9905-1.0430) | 0.2177 |  |  |  |  |  |  |  | | Gender (Female/Male) | 1.3557(0.7785-2.3610) | 0.2823 |  | 1.6268(0.9409-2.8130) | 0.0815 |  |  |  |  |  |  |  | | Treatment (surgery + Adjuvant therapy /surgery) | 1.1402(0.7003-1.8560) | 0.5978 |  | 1.0552(0.6566-1.6960) | 0.8244 |  |  |  |  |  |  |  | | Tumour location (upper, middle, lower) | 1.0694(0.6922-1.6520) | 0.7625 |  | 1.0571(0.6976-1.6020) | 0.7934 |  |  |  |  |  |  |  | | Histologic grade(G1, G2, G3) | 1.0737(0.7264-1.5870) | 0.7214 |  | 0.9449(0.6533-1.3670) | 0.7635 |  |  |  |  |  |  |  | | T stage (T1, T2, T3, T4) | 1.3150(0.4850-3.5670) | 0.5904 |  | 1.3659(0.5201-3.5870) | 0.5268 |  |  |  |  |  |  |  | | N stage (N0, N1, N2, N3) | 2.0583(1.5020-2.8210) | **7.207×10^-06^** |  | 2.0757(1.5370-2.8040) | **1.934×10^-06^** |  |  |  |  |  |  |  | | pTNM stage (I, II, III) | 2.5683(1.6050-4.1090) | **8.356×10^-05^** |  | 2.5737(1.6500-4.0150) | **3.105×10^-05^** |  |  |  |  |  |  |  | | MASAN signature (I, II, III) | 2.0812(1.5390-2.8150) | **1.962×10^-06^** |  | 1.9629(1.4800-2.6030) | **2.856×10^-06^** |  | 1.7200(1.2165-2.4320) | **0.0022** |  | 1.5900(1.0927-2.3130) | **0.0154** |  | | NOTE: Multivariate analysis, Cox proportional hazards regression model. | | | | | | | | | | | |  | |
| --- | --- | --- | --- | --- | --- | --- | --- | --- | --- | --- | --- | --- | --- | --- | --- | --- | --- | --- | --- | --- | --- | --- | --- | --- | --- | --- | --- | --- | --- | --- | --- | --- | --- | --- | --- | --- | --- | --- | --- | --- | --- | --- | --- | --- | --- | --- | --- | --- | --- | --- | --- | --- | --- | --- | --- | --- | --- | --- | --- | --- | --- | --- | --- | --- | --- | --- | --- | --- | --- | --- | --- | --- | --- | --- | --- | --- | --- | --- | --- | --- | --- | --- | --- | --- | --- | --- | --- | --- | --- | --- | --- | --- | --- | --- | --- | --- | --- | --- | --- | --- | --- | --- | --- | --- | --- | --- | --- | --- | --- | --- | --- | --- | --- | --- | --- | --- | --- | --- | --- | --- | --- | --- | --- | --- | --- | --- | --- | --- | --- | --- | --- | --- | --- | --- | --- | --- | --- | --- | --- | --- | --- | --- | --- | --- | --- | --- | --- | --- | --- | --- | --- | --- | --- | --- | --- | --- | --- | --- | --- | --- | --- | --- | --- | --- | --- | --- | --- | --- | --- | --- | --- | --- | --- | --- | --- | --- | --- | --- | --- | --- | --- | --- | --- | --- | --- | --- | --- | --- | --- | --- | --- | --- | --- | --- | --- | --- | --- | --- | --- | --- | --- | --- | --- | --- | --- | --- | --- | --- | --- | --- | --- | --- | --- | --- | --- | --- | --- | --- | --- | --- | --- | --- | --- | --- | --- | --- | --- | --- | --- | --- | --- | --- | --- | --- | --- | --- | --- | --- | --- | --- | --- | --- | --- | --- | --- | --- | --- | --- | --- | --- | --- | --- | --- | --- | --- | --- | --- | --- | --- | --- | --- | --- | --- | --- | --- | --- | --- | --- | --- | --- | --- | --- | --- | --- | --- | --- | --- | --- | --- | --- | --- | --- | --- | --- | --- | --- | --- | --- | --- | --- | --- | --- | --- | --- | --- | --- | --- | --- | --- | --- | --- | --- | --- | --- | --- | --- | --- | --- | --- | --- | --- | --- | --- | --- | --- | --- | --- | --- | --- | --- | --- | --- | --- | --- | --- | --- | --- | --- | --- | --- | --- | --- | --- | --- | --- | --- | --- | --- | --- | --- | --- | --- | --- | --- | --- | --- | --- | --- | --- | --- | --- | --- | --- | --- | --- | --- | --- | --- | --- | --- | --- | --- | --- | --- | --- | --- | --- | --- | --- | --- | --- | --- | --- | --- | --- | --- | --- | --- | --- | --- | --- | --- | --- | --- | --- | --- | --- | --- | --- | --- | --- | --- | --- | --- | --- | --- | --- | --- | --- | --- | --- | --- | --- | --- | --- | --- | --- | --- | --- | --- | --- | --- | --- | --- | --- | --- | --- | --- | --- | --- | --- | --- | --- | --- | --- | --- | --- | --- | --- | --- | --- | --- | --- | --- | --- | --- | --- | --- | --- | --- | --- | --- | --- | --- | --- | --- | --- | --- | --- | --- | --- | --- | --- | --- | --- | --- | --- | --- | --- | --- | --- | --- | --- | --- | --- | --- | --- | --- | --- | --- | --- | --- | --- | --- | --- | --- | --- | --- | --- | --- | --- | --- | --- | --- | --- | --- | --- | --- | --- | --- | --- | --- | --- | --- | --- | --- | --- | --- | --- | --- | --- | --- | --- | --- | --- |

**Table S6. Estimated coefficients for variables used in the MASAN-SI model on OS and DFS.**

| **Variables** | **Mean^a^** | **Coefficients** | |
| --- | --- | --- | --- |
|  |  | OS | DFS |
| **MYC** | 1.0909 | 0.2662 | 0.1640 |
| **ANO1** | 0.0519 | 0.6581 | 0.2697 |
| **SLC52A3** | 0.3636 | 0.2216 | 0.2483 |
| **Age** | 57.3117 | 0.0379 | 0.0293 |
| **N-stage** | 0.9610 | 0.6063 | 0.5293 |

^a^Mean values of variables in the training set, which were used to center the variables when predicting the risk of new patients. OS, Overall survival; DFS, Disease-free survival; MASAN, MYC-ANO1-SLC52A3-Age-N stage

**Table S7. Thresholds of the MASAN-SI grade for OS and DFS**.

| **MASAN-SI grade** | **Risk score range** | |
| --- | --- | --- |
|  | OS | DFS |
| **I** | < -0.4965 | < -0.3077 |
| **II** | -0.4965 ~ 0.2424 | -0.3077 ~ 0.2951 |
| **III** | > 0.2424 | > 0.2951 |

Abbreviation: OS, Overall survival; DFS, Disease-free survival; MASAN, MYC-ANO1-SLC52A3-Age-N stage

**Table S8. OS rate for the MASAN and pTNM staging system in three datasets.**

| Datasets | Stage | MASAN | | | pTNM | | |
| --- | --- | --- | --- | --- | --- | --- | --- |
|  |  | 1-year | 3-year | 5-year | 1-year | 3-year | 5-year |
| Training set | I | 92% | 68% | 64% | 100% | 100% | 100% |
|  | II | 87.5% | 50% | 34.38% | 88.80% | 55.56% | 50% |
|  | III | 55% | 25% | 15% | 71.05% | 39.47% | 23.68% |
| Test set | I | 96.30% | 70.37% | 66.67% | 100% | 80% | 60% |
|  | II | 93.33% | 43.33% | 36.67% | 97.43% | 53.85% | 48.72% |
|  | III | 80% | 20% | 10% | 81.82% | 33.33% | 27.27% |
| Validation set | I | 93.94% | 74.24% | 66.67% | 100% | 50% | 50% |
|  | II | 80.36% | 53.57% | 46.43% | 92.11% | 73.68% | 65.79% |
|  | III | 57.14% | 25% | 21.43% | 70.83% | 40.28% | 34.72% |

**Table S9. DFS rate for the MASAN and pTNM staging system in three datasets.**

| Datasets | Stage | MASAN | | | pTNM | | |
| --- | --- | --- | --- | --- | --- | --- | --- |
|  |  | 1-year | 3-year | 5-year | 1-year | 3-year | 5-year |
| Training set | I | 85.71% | 60.71% | 57.14% | 100% | 100% | 100% |
|  | II | 75.86% | 37.93% | 31.03% | 75% | 47.22% | 44.44% |
|  | III | 45% | 25% | 15% | 65.79% | 34.21% | 23.68% |
| Test set | I | 87.50% | 68.75% | 56.25% | 100% | 80% | 60% |
|  | II | 75.86% | 31.03% | 31.03% | 79.49% | 51.28% | 43.59% |
|  | III | 50% | 0% | 0% | 66.67% | 21.21% | 21.21% |
| Validation set | I | 93.94% | 72.73% | 62.12% | 100% | 50% | 50% |
|  | II | 82% | 52% | 44% | 92.11% | 71.05% | 63.16% |
|  | III | 55.88% | 26.47% | 23.53% | 69.44% | 38.89% | 30.56% |
