# Supplementary figures and images for: MASAN: a novel staging system for prognosis of patients with oesophageal squamous cell carcinoma
Source: Br J Cancer. 2018 May 16;118(11):1476–84. doi: 10.1038/s41416-018-0094-x (PMC5988697; doi:10.1038/s41416-018-0094-x)

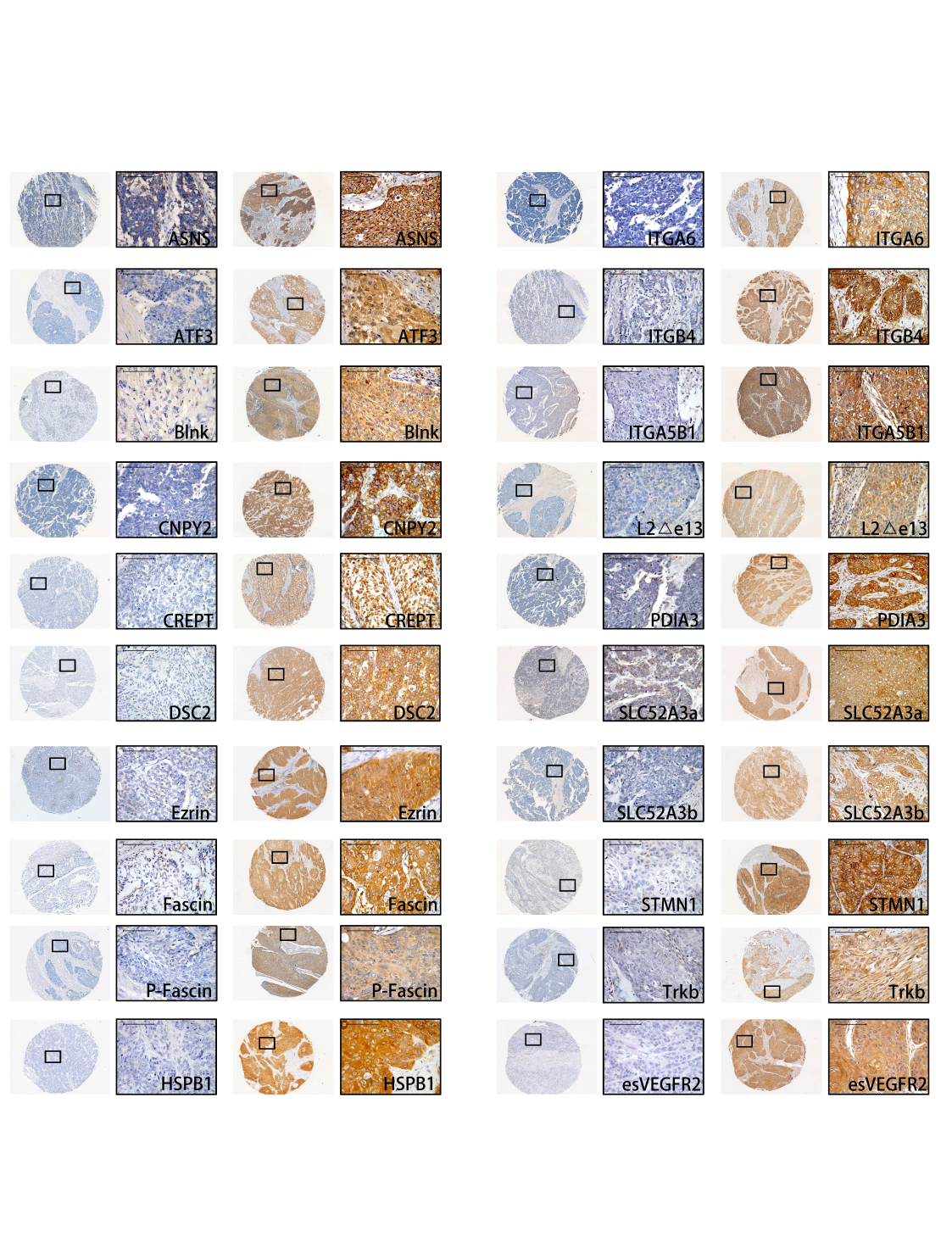

Supplement: Supplementary file 3 — Figure S1 [file 41416_2018_94_MOESM3_ESM.tif]

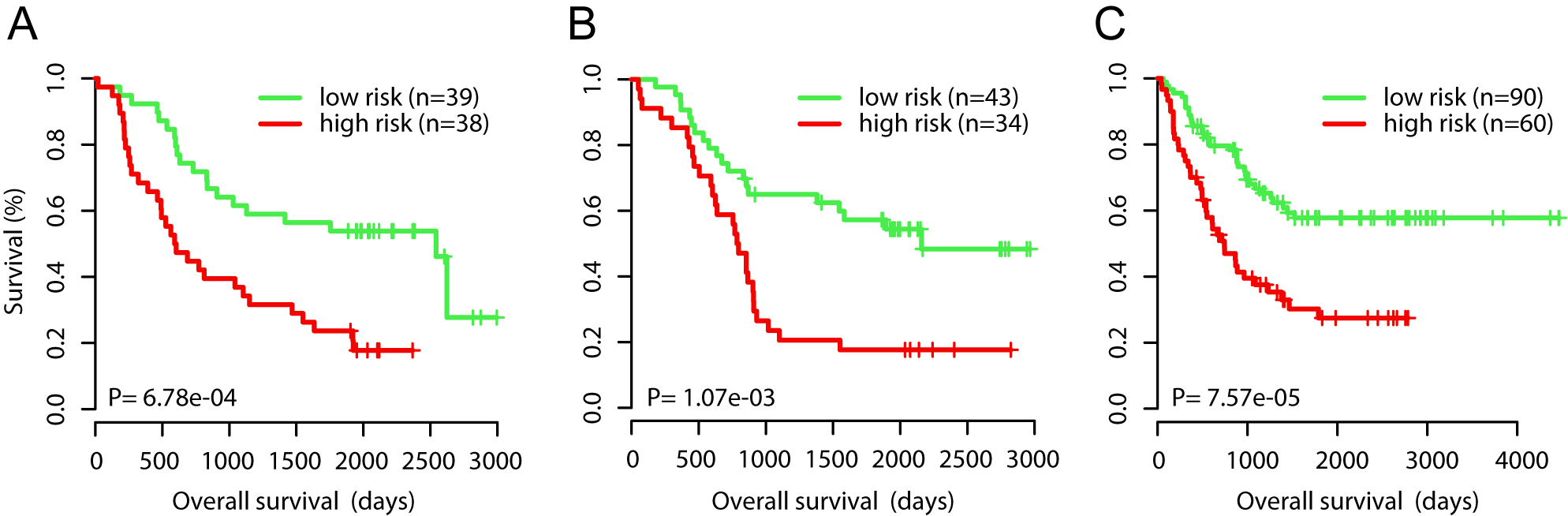

Supplement: Supplementary file 4 — Figure S2 [file 41416_2018_94_MOESM4_ESM.tif]

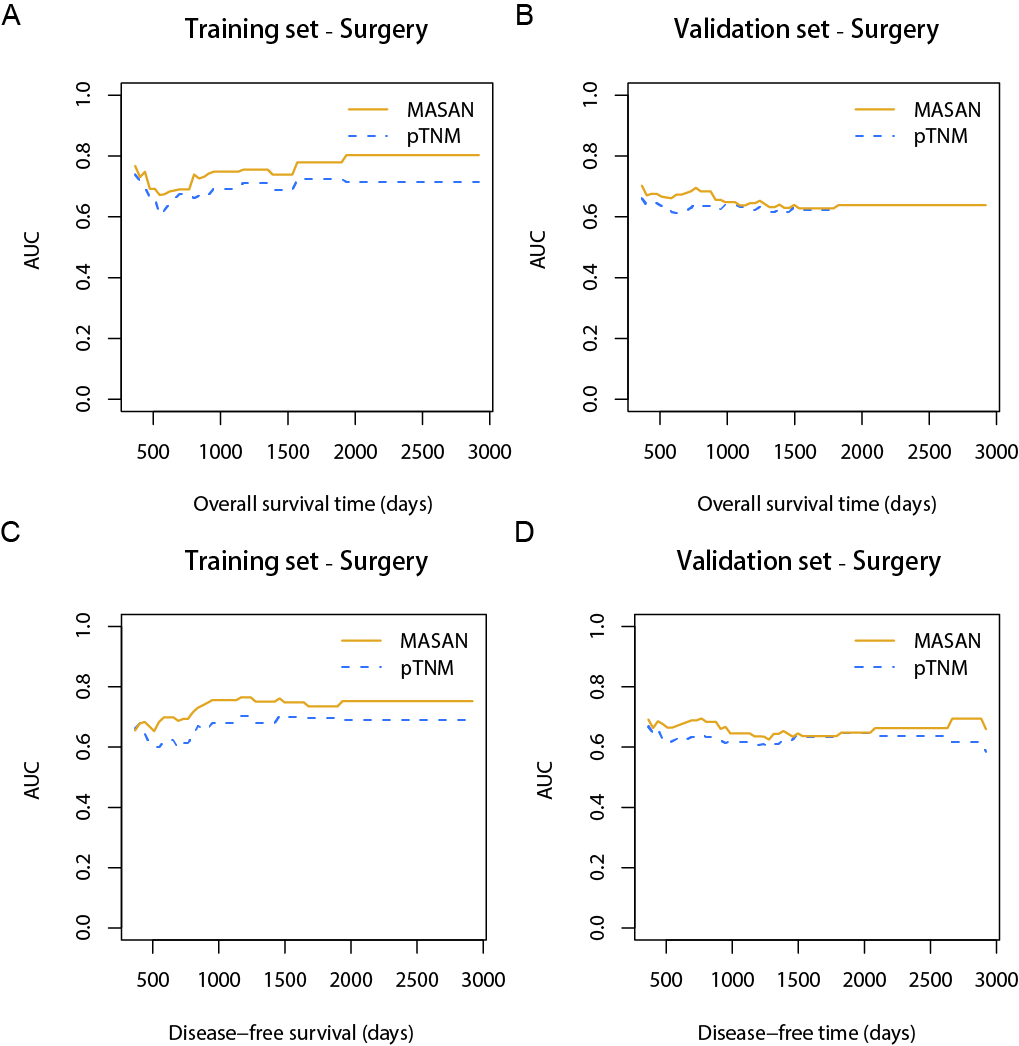

Supplement: Supplementary file 5 — Figure S3 [file 41416_2018_94_MOESM5_ESM.tif]

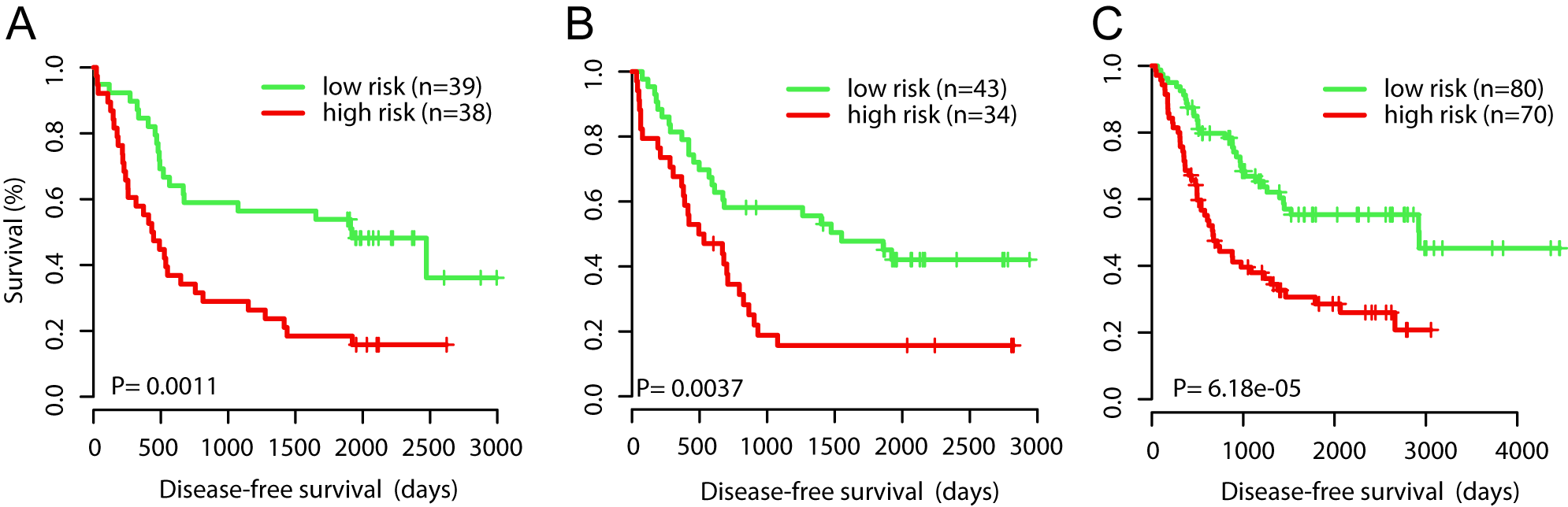

Supplement: Supplementary file 6 — Figure S4 [file 41416_2018_94_MOESM6_ESM.tif]

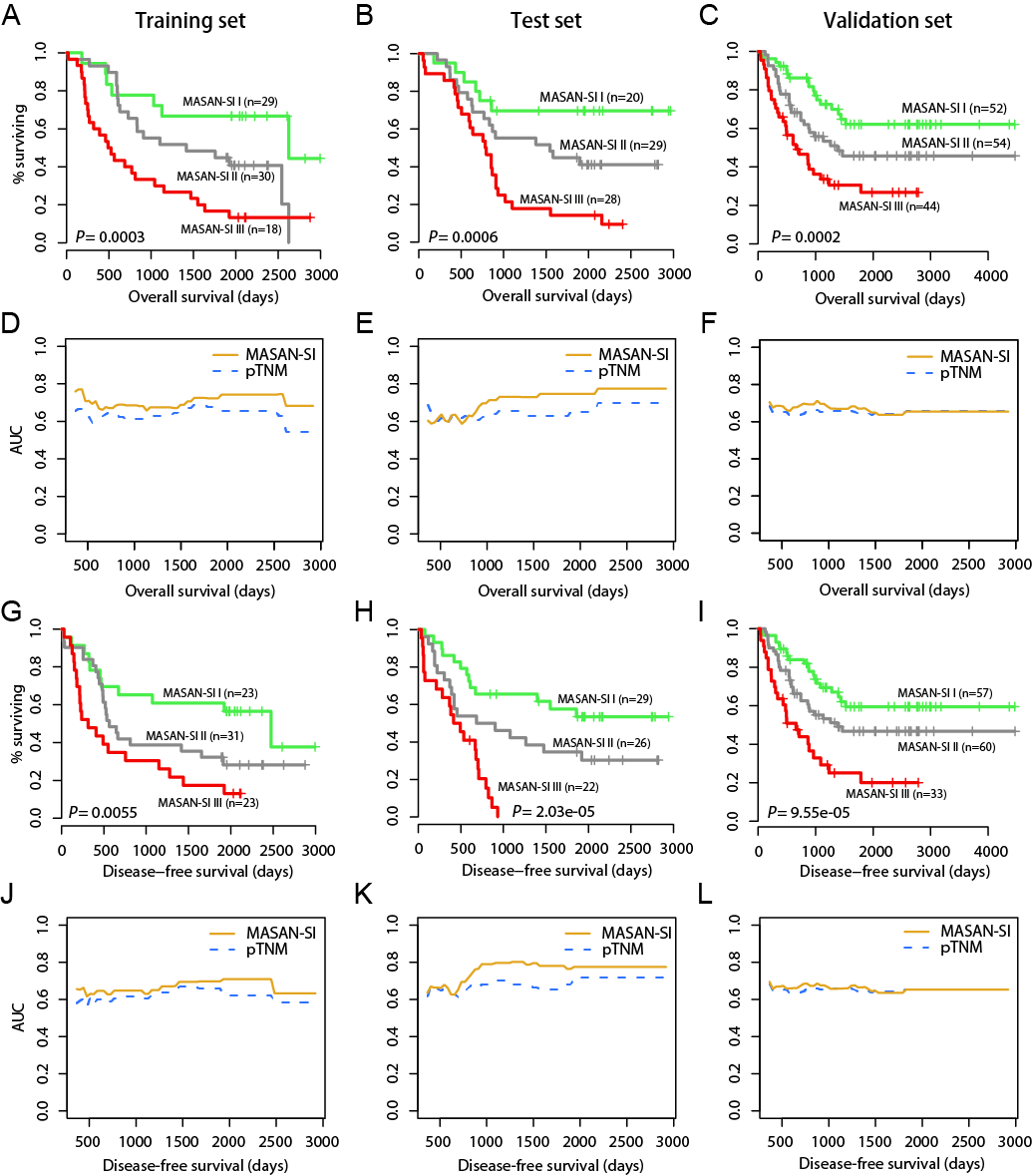

Supplement: Supplementary file 7 — Figure S5 [file 41416_2018_94_MOESM7_ESM.tif]

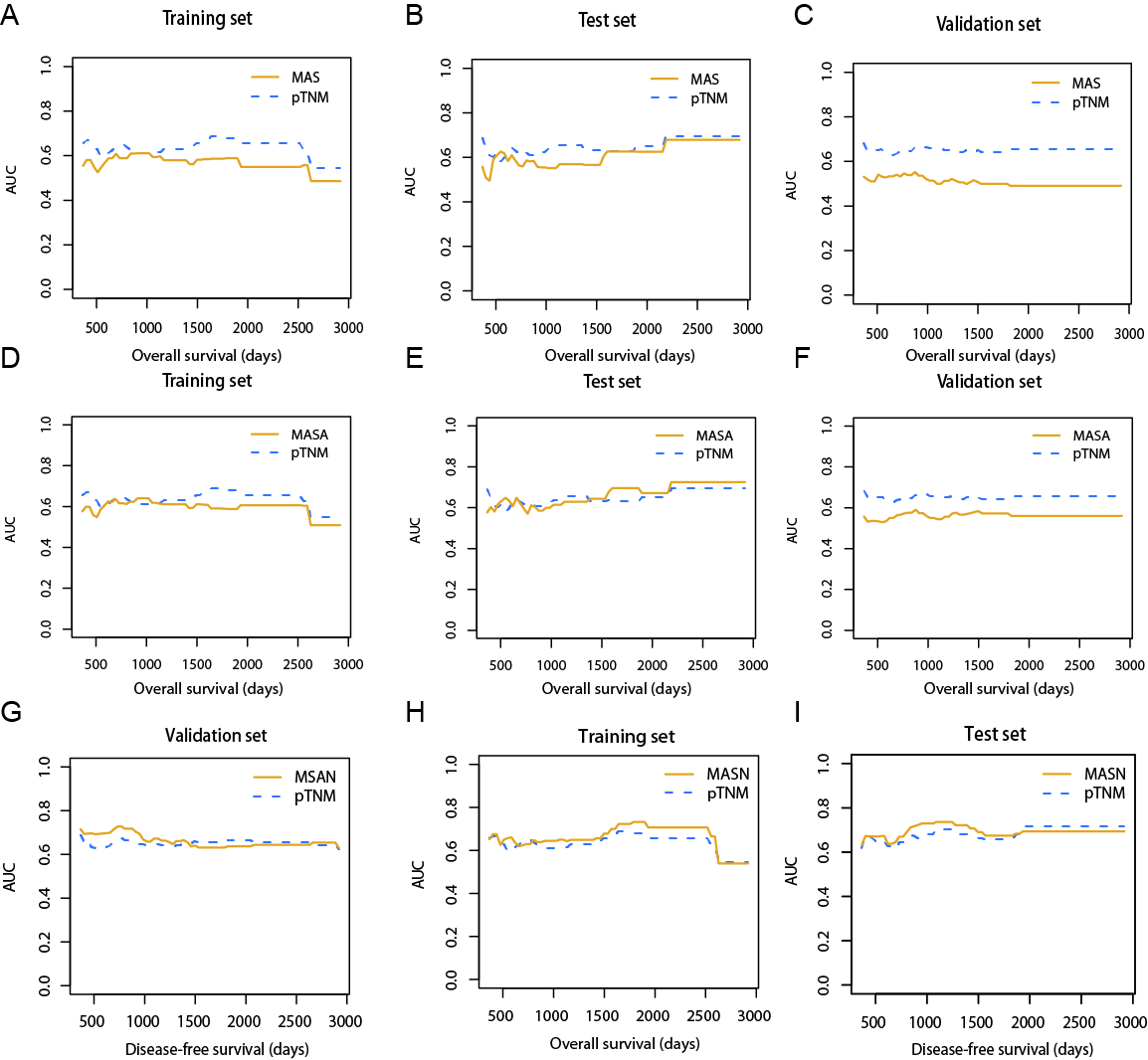

Supplement: Supplementary file 8 — Figure S6 [file 41416_2018_94_MOESM8_ESM.tif]
