# Supplementary material for: MASAN: a novel staging system for prognosis of patients with oesophageal squamous cell carcinoma
Source: Br J Cancer. 2018 May 16;118(11):1476–84. doi: 10.1038/s41416-018-0094-x (PMC5988697; doi:10.1038/s41416-018-0094-x)
Supplement: Supplementary file 9 — Supplementary Figures [file 41416_2018_94_MOESM9_ESM.docx]

**Figure S1.** Representative images of immunohistochemical staining for ASNS, ATF3, Blnk, CNPY2, CREPT, DSC2, ezrin, fascin, p-fascin, HSPB1, ITGA6, ITGB4, ITGA5B1, L2△e13, PDIA3, SLC52A3a, SLC52A3b, STMN1, esVEGFR2, and Trkb in tissue microarrays. (Scale bars=50 μm)

**Figure S2. Kaplan–Meier curves for OS of ESCC patients stratified into low-risk and high-risk groups.** (**A**) Training set (*n*=77). (**B**) Test set (*n*=77). (**C**) Validation set (*n*=150).

**Figure S3. Prognostic performance of MASAN on patients with surgery alone.** (**A**, **B**) The time-dependent AUCs of the MASAN and pTNM staging systems on OS for the new training set (37 in the training set and 39 in the test set) (**A**) and validation set (*n*=131) (**B**). (**C**, **D**) The time-dependent AUCs of the MASAN and pTNM staging systems on DFS for the new training set (**C**) and validation set (**D**).

**Figure S4. Kaplan–Meier curves for DFS of ESCC patients stratified into low-risk and high-risk groups.** (**A**) Training set (*n*=77). (**B**) Test set (*n*=77). (**C**) Validation set (*n*=150).

**Figure S5. Predictive performance of MASAN-SI.** (**A**-**C**) Kaplan-Meier curves using the MASAN-SI system on OS for the training set (**A**), test set (**B**) and validation set (**C**). (**D**-**F**) Time-dependent AUCs using the MASAN-SI and pTNM staging systems on OS for the training set (**D**), test set (**E**) and validation set (**F**). (**G**-**I**) Kaplan-Meier curves using MASAN-SI on DFS for the training set (**G**), test set (**H**) and validation set (**I**). (**J**-**L**) Time-dependent AUCs using the MASAN-SI and pTNM staging systems on DFS for the training set (**J**), test set (**K**) and validation set (**L**). *P*-values were calculated by the log-rank test.

**Figure S6. All the five MASAN features are essential.** (**A-C**) The time-dependent AUCs using the MAS and pTNM staging systems on the OS for the training set (**A**), test set (**B**), and validation set (**C**). MSA, MASAN model without age and N-stage. (**D-F**) The time-dependent AUCs using the MASA and pTNM staging systems on the OS for the training set (**D**), test set (**E**), and validation set (**F**). MASA, MASAN model without N-stage. (**G**) The time-dependent AUCs using MSAN and pTNM staging systems on DFS for the validation set. MSAN: MASAN model without ANO1. (**H**,**I**) The time-dependent AUCs using the MASN and pTNM staging systems on the OS for the training set (**H**) and validation set (**I**). MASN, MASAN model without Age.
